# Engineering yeast with a light-driven proton pump system in the vacuolar membrane

**DOI:** 10.1186/s12934-023-02273-1

**Published:** 2024-01-03

**Authors:** Kaoru M. Daicho, Yoko Hirono-Hara, Hiroshi Kikukawa, Kentaro Tamura, Kiyotaka Y. Hara

**Affiliations:** 1 Graduate Division of Nutritional and Environmental Sciences, University of Shizuoka, 52-1 Yada, Suruga-Ku, Shizuoka, 422-8526 Japan; 2 396Bio, Inc., University of Shizuoka, 52-1 Yada, Suruga-Ku, Shizuoka, 422-8526 Japan; 3 Department of Environmental and Life Sciences, School of Food and Nutritional Sciences, University of Shizuoka, 52-1 Yada, Suruga-Ku, Shizuoka, 422-8526 Japan

**Keywords:** Vacuole, Rhodopsin, Light, ATP, Yeast, Engineering biology

## Abstract

**Background:**

The supply of ATP is a limiting factor for cellular metabolism. Therefore, cell factories require a sufficient ATP supply to drive metabolism for efficient bioproduction. In the current study, a light-driven proton pump in the vacuolar membrane was constructed in yeast to reduce the ATP consumption required by V-ATPase to maintain the acidification of the vacuoles and increase the intracellular ATP supply for bioproduction.

**Results:**

Delta rhodopsin (dR), a microbial light-driven proton-pumping rhodopsin from *Haloterrigena turkmenica*, was expressed and localized in the vacuolar membrane of *Saccharomyces cerevisiae* by conjugation with a vacuolar membrane-localized protein. Vacuoles with dR were isolated from *S. cerevisiae*, and the light-driven proton pumping activity was evaluated based on the pH change outside the vacuoles. A light-induced increase in the intracellular ATP content was observed in yeast harboring vacuoles with dR.

**Conclusions:**

Yeast harboring the light-driven proton pump in the vacuolar membrane developed in this study are a potential optoenergetic cell factory suitable for various bioproduction applications.

**Supplementary Information:**

The online version contains supplementary material available at 10.1186/s12934-023-02273-1.

## Background

The ATP supply in microorganisms is a major limiting factor for cellular metabolism. Therefore, increasing the ATP supply may improve microbial production systems that use cellular metabolism to manufacture useful compounds [1]. Attempts to increase the ATP content in cells have included: (1) the addition of energy substrates; (2) controlling the pH; (3) metabolic engineering of ATP-generating or ATP-consuming pathways; and (4) controlling the reactions of the respiratory chain. Recently, light-driven ATP supply using rhodopsin, a light-driven proton pump protein with all-*trans*-retinal as a chromophore, has also been investigated [2].

Bacterial proteorhodopsin has been expressed in *Escherichia coli* to generate a proton motive force that can directly drive a proton-driven flagellar motor [3] and hydrogen production [4]. Moreover, a light-driven proton pump has been introduced to couple with proton-driven F_o_F_1_-ATP synthase in *E. coli* [5]. Inside-out cell membrane vesicles generated from *E. coli* expressing delta rhodopsin (dR) from *Haloterrigena turkmenica* have been used to supply light-driven ATP for cell-free bioproduction [5]. Bacteriorhodopsin from *Halobacterium salinarum* has been reconstructed into liposomes and coupled with the ribosome system for protein biosynthesis [6]. Recently, *E. coli* expressing dR has been directly used as a prokaryotic synthetic bioengineering host strain that could enhance ATP supply in producing several useful compounds [2, 7].

In eukaryotes, dR has been expressed in the mitochondria of mammalian cells [8] and *Drosophila* [9] to increase the intracellular ATP supply. Therefore, the light-driven proton pumping activity of microbial rhodopsin is also a promising way to solve the fundamental energy limitation in eukaryotic cell factories. The budding yeast *Saccharomyces cerevisiae* has been used as a eukaryotic bioengineering host strain for the bioproduction of various compounds. Therefore, *S. cerevisiae* harboring the light-driven proton pump rhodopsin has potential as an attractive host strain for various industrial bioproduction applications. In *S. cerevisiae*, maintaining acidification inside the vacuoles consumes large amounts of ATP to pump protons from the cytosol to the vacuolar lumen by the vacuolar ATPase (V-ATPase), which is localized in vacuolar membranes [10, 11].

**Fig. 1:**
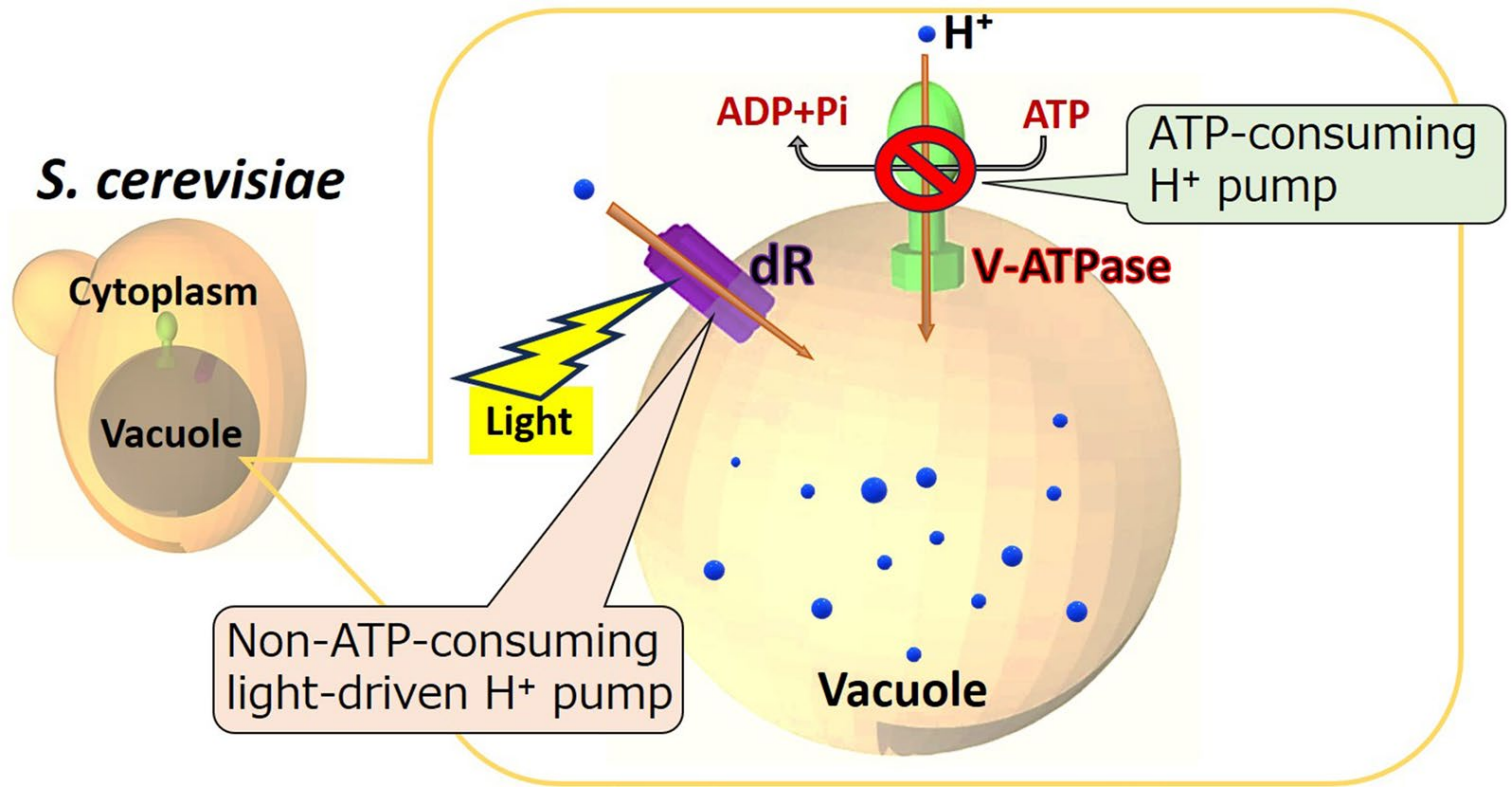
Schematic illustration of the concept of this study. In *S. cerevisiae*, acidic organelle vacuoles occupy a large part of the intracellular volume. The ATP-consuming proton pump V-ATPase constantly translocates H^+^ from the cytosol to the vacuolar lumen to maintain the acidification of vacuoles. Reducing the amount of ATP consumed by V-ATPase should increase the ATP supply for the bioproduction of valuable compounds in *S. cerevisiae*. In this study, the non-ATP-consuming light-driven H^+^ pump dR was introduced into the vacuolar membrane. The introduced dR translocates H^+^ from the cytosol to the vacuolar lumen under light conditions

In the present study, we developed optoenergetic *S. cerevisiae* by introducing dR into the vacuolar membrane (Fig. 1). Light-driven proton pumping by the introduced rhodopsin was hypothesized to replace ATP-driven proton pumping via the hydrolysis of ATP by V-ATPase on vacuoles in *S. cerevisiae*. The light-driven proton pumping by dR from the outside to the inside of vacuoles was observed as decreases in the proton concentration, calculated from the pH outside of the isolated vacuoles. Furthermore, the intracellular ATP content was increased in engineered *S. cerevisiae* harboring vacuoles expressing dR by light irradiation. The optoenergetic yeast constructed in this study can potentially solve the fundamental energy limitation problem of cell factories for various bioproduction applications.

## Results

### Localization of dR in the vacuolar membrane

**Fig. 2:**
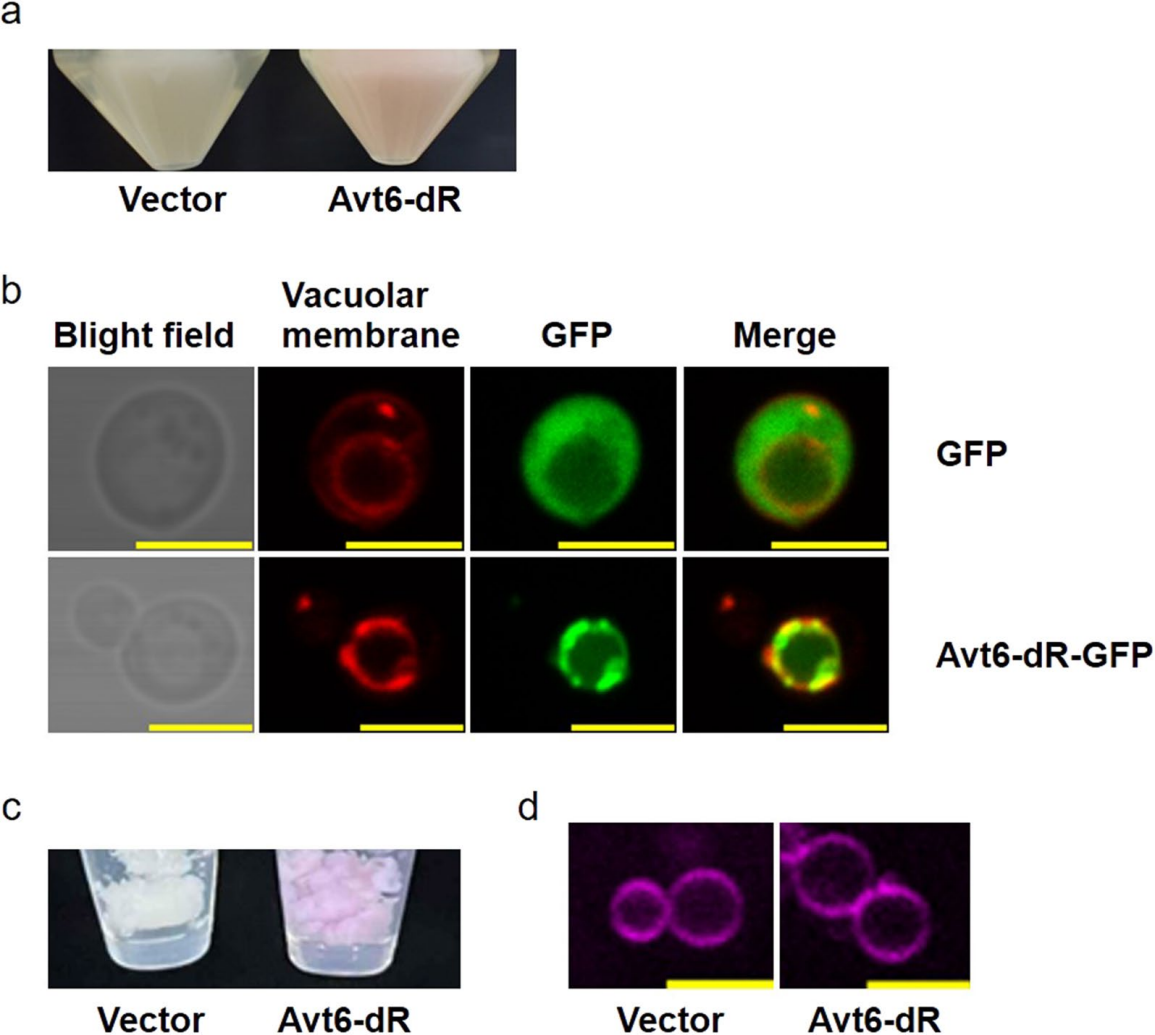
Observations of *S. cerevisiae* cells and isolated vacuoles. **a** Cell pellet of the vector control strain and the strain expressing Avt6-dR supplemented with all-*trans*-retinal. **b** Fluorescence microscopy images of cells expressing GFP (upper panels) and Avt6-dR-GFP (lower panels). The vacuolar membrane was labeled with FM4-64. **c** Vacuoles isolated from the vector control strain and the strain expressing Avt6-dR. **d** Fluorescence microscopy images of vacuoles isolated from the vector control strain and the strain expressing Avt6-dR. The vacuolar membrane was labeled with FM4-64 (magenta). Each scale bar (yellow line) represents 5 µm

The amino acid transporter Avt6 is a vacuolar membrane-localized protein [12]. The *C*-terminus of Avt6 was conjugated at the *N*-terminus of dR to localize dR in the vacuolar membrane with the direction of pumping protons from the cytosol to the vacuolar lumen. The expression of Avt6-dR in the vacuolar membrane of *S. cerevisiae* resulted in cells turning pink after adding the all-*trans*-retinal. In contrast, the vector control strain was beige, which is the natural color of *S. cerevisiae*, even when the all-*trans*-retinal was present (Fig. 2a). To confirm the localization of Avt6-dR in the vacuolar membrane of *S. cerevisiae*, Avt6-dR conjugated to green fluorescent protein (GFP) was expressed in *S. cerevisiae*. The vacuoles were labeled specifically by the red fluorescent dye FM4-64 [13]. Fluorescent observation of unconjugated GFP showed diffusion of GFP in the cytosol of the yeast (upper panels of Fig. 2b). In contrast, Avt6-dR-GFP was localized in the vacuolar membrane as shown by the overlap of FM4-64 in the vacuolar membranes with the green fluorescence from GFP conjugated with Avt6-dR (lower panels of Fig. 2b).

The vacuoles expressing Avt6-dR and the vector control vacuoles were isolated using the Ficoll method [14] from the *S. cerevisiae* strain expressing Avt6-dR and the vector control strain, respectively, after culture with all-*trans*-retinal. The isolated vacuoles expressing Avt6-dR were pink, and the vector control vacuoles were white (Fig. 2c). The isolated vacuoles dissolved in buffer with sucrose were completely spherical, and there was no difference between the size of vacuoles with or without dR (Fig. 2d), which were the same shape and size as when localized inside the intact cells (Fig. 2b).

### Light-driven proton pumping activity of yeast vacuoles expressing dR

**Fig. 3:**
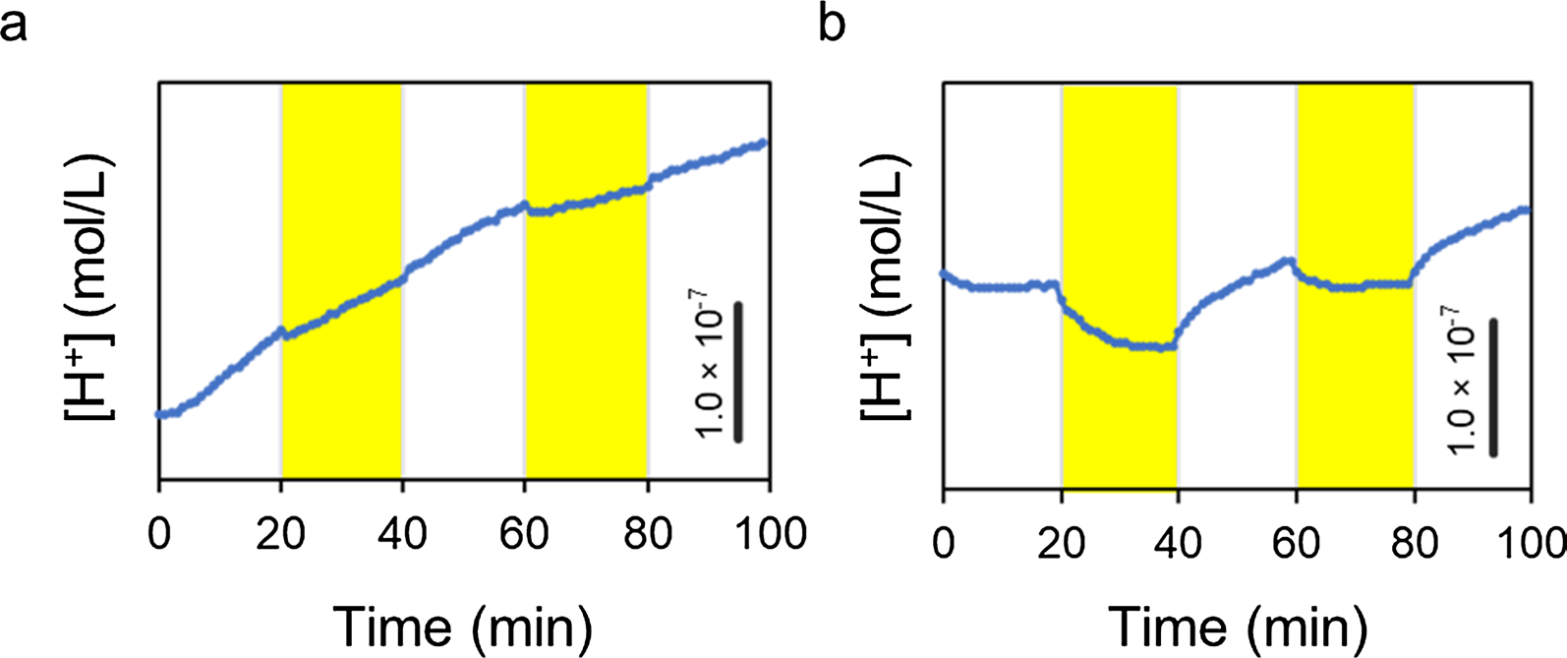
Time course of the proton concentration converted from the pH value. **a** Time course of the proton concentration with the vacuoles isolated from the vector control strain, with the light switched on and off. **b** Time course of the proton concentration with the vacuoles isolated from the strain expressing dR, with the light switched on and off. The bars indicate the differences in the proton concentration. The yellow area indicates the light-irradiated period at 100 µmol photons/m^2^/s

To confirm the effect of dR localization in the vacuolar membrane, vacuoles with or without dR were isolated and their light-driven proton pumping activities were compared. The pH changes in the vacuole suspensions were monitored during switching between dark and light conditions at 20-min intervals. The monitored pH was transformed to a proton concentration and is shown in Fig. 3. The proton concentration of the suspension containing the isolated vacuoles without dR constantly increased under light irradiation, similar to that under dark conditions (Fig. 3a). In contrast, the proton concentration of the suspension containing the isolated vacuoles with dR decreased under light irradiation (Fig. 3b). This reduction in the proton concentration indicated the light-driven proton pumping activity of the vacuoles with dR.

### The effect of vacuolar dR expression and light irradiation on cell growth and intracellular ATP supply

**Fig. 4:**
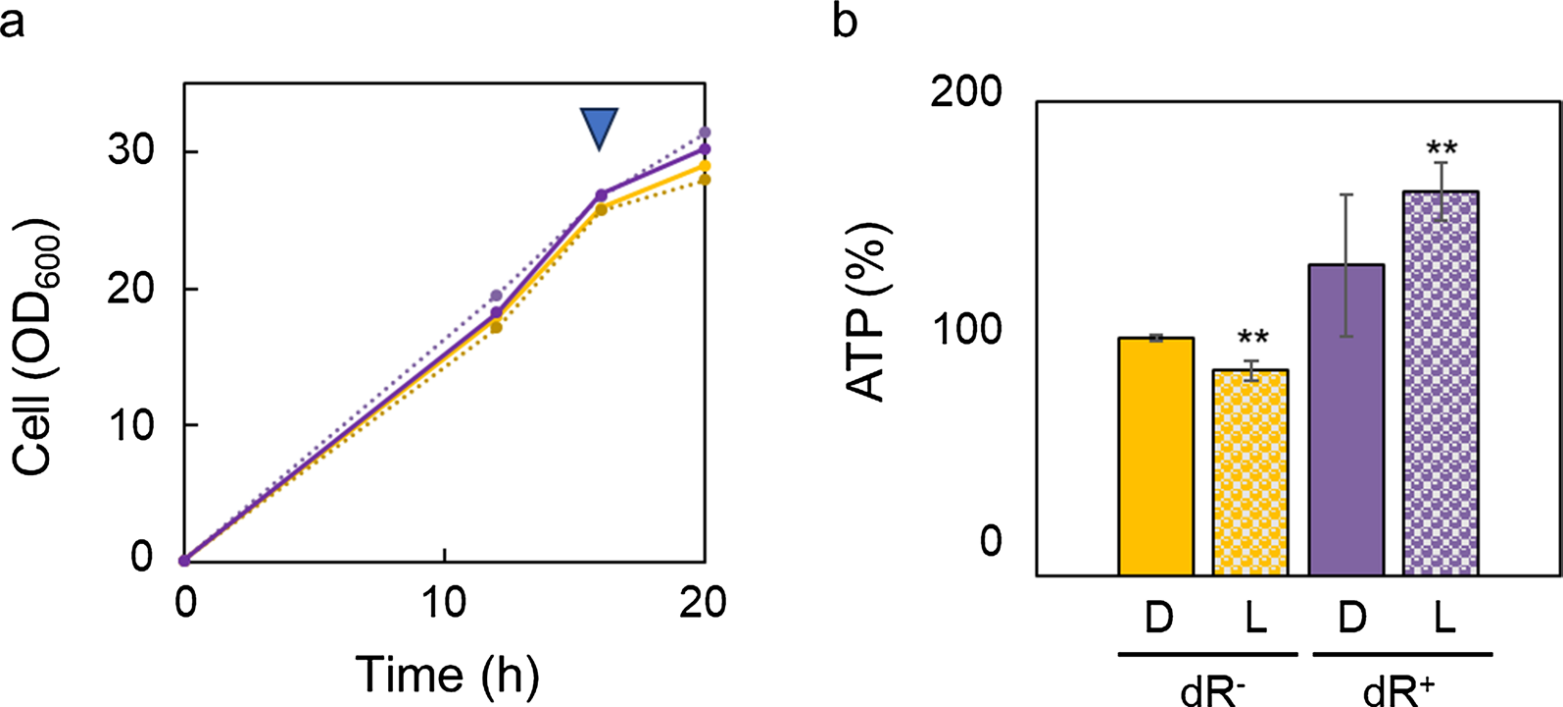
Effect of dR expression and light irradiation on cell growth and intracellular ATP content. *S. cerevisiae* harboring vacuoles without and with dR were cultured under dark (5 µmol photons/m^2^/s) and light (100 µmol photons/m^2^/s) conditions. **a** Time course of the cell concentration (OD_600_). The yellow and blue lines indicate the yeast harboring vacuoles without and with dR, respectively. The solid and dotted lines indicate cultivation under dark and light conditions, respectively. The triangle indicates the sampling point at 16 h for ATP measurement. **b** The relative intracellular ATP content extracted from the yeast harboring vacuoles without and with dR. “D” and “L” indicate cultivation under dark and light conditions, respectively. Means and standard deviations are shown (*n* = *3*). Double asterisks represent significant differences compared with yeast harboring vacuoles without dR under dark conditions in the *t*-test (p < 0.01)

The effect of the light-driven proton pump in the vacuolar membrane on ATP supply was evaluated by investigating the effect of dR expression in the vacuolar membrane of *S. cerevisiae* on cell growth and intracellular ATP content. The concentrations of the cells (OD_600_) of the strain expressing dR and the vector control strain were measured during cultivation under dark (5 µmol photons/m^2^/s) and light (100 µmol photons/m^2^/s) conditions (Fig. 4a). Vacuolar dR expression and the light irradiation did not influence the cell growth of *S. cerevisiae*. The intracellular ATP content in the two strains was compared after cultivation for 16 h (Fig. 4b). The results showed that light irradiation of the vector control strain decreased the intracellular ATP content by approximately 0.86-fold. In contrast, light irradiation of the strain expressing dR in the vacuolar membrane increased the intracellular ATP content by approximately 1.6-fold compared with the vector control strain. Furthermore, as shown in Additional file 1: Fig. S1, cell growth and ATP levels of *S. cerevisiae* with or without dR after 18 h of cultivation at pH 7 and 4 were compared under a light (100 µmol photons/m^2^/s) condition. The results showed that the expression of dR in the vacuolar membrane did not affect cell growth at pH 7 and 4; however, dR expression elevated the ATP level at pH 4 by ~ 1.5-fold compared with that at pH 7.

## Discussion

We engineered vacuoles in a budding yeast that consumes a large amount of ATP to maintain internal acidification [10, 11]. This acidification maintenance using an ATP-hydrolyzing proton pump is performed by V-ATPase localized in the vacuolar membrane. In the present study, we artificially localized the light-driven proton pump dR in the vacuolar membrane by fusing dR with the vacuolar membrane-localized protein Avt6. We hypothesized that if light-driven proton pumping by dR could replace ATP-hydrolyzing proton pumping by V-ATPase, it would be possible to reduce the ATP consumption by V-ATPase and thus increase the intracellular ATP supply. As shown in Fig. 3, the results indicated that light-driven proton pumping activity from outside to inside the vacuole by dR in the vacuolar membranes was successfully observed (Fig. 3b), and this activity was not observed when vacuoles without dR were used (Fig. 3a). The proton concentration continuously increased in the suspension containing vacuoles without dR (Fig. 3a). This increase in the proton concentration indicated that proton leakage from inside the vacuole through the membrane had occurred. This proton leakage would have occurred through the action of a vacuolar Na^+^(K^+^)/H^+^ antiporter and Ca^2+^/H^+^ antiporters [15]. In contrast, the proton concentration was stable in the suspension containing vacuoles with dR before light irradiation (Fig. 3b), although proton leakage was observed after a higher proton concentration inside the vacuoles was generated by dR with light irradiation. These results indicated that the protons taken into the vacuoles by dR during light irradiation leaked from the vacuoles through the action of a vacuolar Na^+^(K^+^)/H^+^ antiporter and Ca^2+^/H^+^ antiporters. Furthermore, in yeast cells, cation transporters in the plasma membrane, such as Na^+^(K^+^)/H^+^ antiporters and H^+^-ATPase, also contribute significantly to maintaining cytosolic pH and ATP levels [16]. Therefore, the activity of V-ATPase, which translocates protons from the cytosol to the vacuolar lumen, is influenced by these cation transporters in the plasma membrane. Thus, the activity of dR expressed in the vacuolar membrane would also be influenced by these cation transporters in the plasma membrane.

In previous studies, increases in intracellular ATP supply were achieved by expressing dR in the mitochondria of eukaryotic cells, such as mammalian cells [8] and *Drosophila* [9]. A trial of the expression of rhodopsin in the vacuoles of *S. cerevisiae* was preliminarily reported in a patent in 2021 [17] and a preprint in 2023 [18]; however, the effect of this expression in vacuoles and cellular energetic metabolism has not been reported previously. This study is the first report describing the direct measurement of light-driven proton pumping activity from isolated vacuoles expressing bacterial rhodopsin (Fig. 3b). This is also the first study to report light-dependent increases in ATP (Fig. 4b) in yeast harboring vacuoles expressing dR, without growth defects (Fig. 4a). Light irradiation at 100 µmol photons/m^2^/s of the yeast expressing dR in the vacuolar membrane increased the intracellular ATP content by approximately 1.6-fold (Fig. 4b) compared with the vector control strain. We have previously reported that light irradiation at 100 µmol photons/m^2^/s of *E. coli* expressing dR in the cytosolic membrane increased the intracellular ATP content by approximately 1.5-fold compared with the vector control strain [2]. We also showed that light irradiation of this *E. coli*-expressing dR enhanced the production of some valuable compounds such as glutathione, mevalonate, 3-hydroxypropionate [2] and isoprenol [7]. The yeast harboring the light-driven proton pump in the vacuolar membrane developed herein also has potential as an optoenergetic cell factory for various bioproduction applications because yeast is used as a host strain for metabolically engineered cell factories, and ATP supply is the rate-limiting step in many bioproduction processes [1].

## Conclusions

The light-driven proton-pumping bacterial rhodopsin dR from *H. turkmenica* was expressed in the vacuolar membrane in *S. cerevisiae*. The vacuoles with dR were isolated and light-driven proton pumping activity was confirmed. A light-dependent increase in the ATP content was observed in the yeast harboring vacuoles with dR. This result indicated that light-driven proton pumping into vacuoles by dR partially replaced ATP-consuming proton pumping by V-ATPase, which maintains the acidification of vacuoles in yeast. The yeast harboring the light-driven proton pump in the vacuolar membrane may be a powerful host strain for synthetic bioengineering and metabolic engineering to produce various valuable target products.

## Methods

### Strains and media

DH5α (Nippon Gene, Tokyo, Japan) was used as the *E. coli* host strain for recombinant DNA manipulation. *E. coli* transformants were grown in Luria–Bertani medium (10 g/L tryptone, 5 g/L yeast extract, and 10 g/L sodium chloride) supplemented with 100 μg/ml ampicillin. *S. cerevisiae* BY4741 [*MATa his3-Δ1 leu2-Δ0 met15-Δ0 ura3-Δ0*] was used as the yeast parental strain for gene expression. Yeast transformants were cultured in synthetic defined (SD) medium (6.7 g/L yeast nitrogen base w/o amino acids, and 20 g/L d-glucose) or yeast extract–peptone–dextrose (YPD) medium (20 g/L tryptone, 10 g/L yeast extract, and 20 g/L d-glucose). Amino acids (20 mg/L histidine, 60 mg/L leucine, and 20 mg/L methionine) were supplemented into the SD media lacking the relevant auxotrophic components. Tryptone, yeast extract, and yeast nitrogen base w/o amino acids were purchased from Becton Dickinson Japan (Tokyo, Japan). Other chemicals were obtained from Nacalai Tesque (Kyoto, Japan) or Fujifilm Wako Chemicals (Osaka, Japan).

### Plasmid construction and yeast transformation

The nucleic acid sequences in the chromosomal DNA of *H. turkmenica* (accession No: JCM 9743) for codon-optimized dR for *S. cerevisiae* were designed and synthesized. The forward and reverse primers used for the amplification in the cloning were pGK426-dR_F (5′-GTCGACACTAGTGGATCCCCCGGGATGTGCTGTGCTGCTTTGG-3′) and dR-pGK426_R (5′-AGATCTGAATTCTCTAGACCCTCAGGTTGGAGCAGCTGTAGGAG-3′), respectively. The amplified fragment was inserted into a pGK426 vector [19] and digested with *Sma*I to construct pGK426-dR using an In-Fusion HD Cloning Kit (Takara Bio, Shiga, Japan). The enhanced GFP gene with codon usage optimized for expression in *S. cerevisiae* [20] was synthesized, digested by *Eco*RV and *Eco*RI, and inserted into the corresponding restriction site of pGK426-*dR* to create pGK426-*dR*-*GFP*. The gene *AVT6* encoding the vacuolar membrane-localized protein Avt6 was amplified by PCR from the genome of the *S. cerevisiae* BY4741 strain using the primers pGK426-*AVT6*_F (5′-GTCGACACTAGTGGATCCCCCGGGATGGTAGCTAGTATTAGATCAGGTGTGC-3′) and *AVT6*-dR_R (5′-CAAAGCAGCACAGCACATCCCGTTTAGCTTCAAAGCTGCGGTCAG-3′). The amplified fragment was inserted into pGK426-*dR* and pGK426-*dR*-*GFP* and digested with *Sma*I to construct pGK426-*AVT6*-*dR* and pGK426-*AVT6-dR-GFP*, respectively. The plasmids pGK426-*GFP*, pGK426-*AVT6*-*dR*, and pGK426-*AVT6-dR-GFP* and the vector pGK426 were transformed into the *S. cerevisiae* BY4741 strain using a lithium acetate method as previously described [21, 22] to construct strains expressing *GFP*, *AVT6*-*dR*, and *AVT6-dR-GFP* and the vector control strain, respectively. All synthetic genes and primers were purchased from Eurofins Genomics (Tokyo, Japan).

### Isolation of vacuoles from *S. cerevisiae*

Vacuoles were isolated from *S. cerevisiae* according to a previous report [14] with some modifications. The glycerol stock of *S. cerevisiae* was cultured in SD (His, Leu, and Met) medium on a plate. Three colonies were selected, inoculated into YPD medium, and cultured at 30 °C with agitation at 175 rpm for 15.5 h in a shaking incubator, BR-43FL (TAITEC, Saitama, Japan). Cultured *S. cerevisiae* was inoculated into 200 mL of fresh YPD medium with 10 µM all-*trans*-retinal (Sigma‒Aldrich, St. Louis, MO, USA) at an OD_600_ value of 0.15 and cultured at 30 °C with agitation at 175 rpm for 15 h. The cells were collected by centrifugation [1200×*g*, 2 min, room temperature (RT)] and suspended in 20 mL of TD buffer [0.1 M Tris–HCl (pH 8.0), and 10 mM DTT]. The weight of the cells after centrifugation (1200×*g*, 2 min, RT) was measured, and the cells were inoculated into 20 mL of YSZ solution (10 g/L tryptone, 5 g/L yeast extract, 10 g/L d-glucose, 1.0 M d-sorbitol, and Zymolyase-100 T). The weight of added Zymolyase-100 T was 1% of the wet cell weight. The cell walls were lysed to generate spheroplasts by gentle mixing (100 rpm, 1 h, 30 °C) of the cell suspension. Then, 35 mL of spheroplast suspension was mixed with 20 mL of HS buffer [20 mM HEPES–KOH (pH 7.2) and 1.2 M d-sorbitol]. Spheroplasts collected by centrifugation (500×*g*, 5 min, RT) were inoculated into 40 mL of YPS solution (10 g/L tryptone, 5 g/L yeast extract, 10 g/L d-glucose, and 1.0 M d-sorbitol) and incubated at 30 °C with agitation at 100 rpm for 2.5 h. After cooling the suspension on ice for 5 min, the spheroplasts were collected by centrifugation (500×*g*, 5 min, 4 °C). The collected spheroplasts were dissolved in 6 mL of 12% FPS solution [10 mM PIPES-KOH (pH 6.4), 0.2 M d-sorbitol, and 12% Ficoll PM400]. Samples were filtered through a TMTP02500 membrane filter (Merck Millipore, Burlington, MA, USA). A Ficoll step gradient solution was constructed by adding 100 µL of 20% FPS solution [10 mM PIPES-KOH (pH 6.4), 0.2 M d-sorbitol, and 20% Ficoll PM400], 600 µL of sample dissolved in a 12% FPS solution, and 400 µL of an 8% FPS solution [10 mM PIPES-KOH (pH 6.4), 0.2 M d-sorbitol, and 8% Ficoll PM400] from the bottom to the top of the ultracentrifugation tube. After ultracentrifugation (33,000×*g*, 30 min, 4 °C), the vacuole fraction was obtained from around the top of the 8% FPS solution and stored at –80 °C until use.

### Measurement of the proton pumping activity of vacuoles

To evaluate the proton pumping activity of the vacuoles, vacuoles isolated from *S. cerevisiae* were used for ∆pH measurements [2, 23]. After isolation, the vacuoles were washed three times with SS-HCl solution [10 mM NaCl, 10 mM MgSO_4_·7H_2_O, 0.1 mM CaCl_2_·2H_2_O, 1.0 M d-sorbitol, and 1.0 mM HCl (pH 4.4)] and resuspended in the same solution.

The light-driven proton pumping activity of the vacuoles was measured by monitoring changes in the pH of the vacuole suspension. The suspension was illuminated using a 300 W halogen projector lamp (JCD100V-300 W, CABIN CS-30AF) through a bandpass filter (PBO530-120, Asahi Spectra, Tokyo, Japan) at a wavelength of 530 ± 120 nm. The intensity of the light irradiation of the samples was adjusted to 100 µmol photons/m^2^/s using an LA-105 light analyzer (NK system, Osaka, Japan). The pH values of the vacuole suspensions were monitored over time and logged using a pH meter (F-72, Horiba, Kyoto, Japan).

### Microscopic observations of *S. cerevisiae* vacuoles

The localization of Avt6-dR was observed using a fluorescence microscope. *S. cerevisiae* BY4741/*AVT6*-*dR*-*GFP* precultured in YPD was cultured in 20 mL of SD (His, Leu, and Met) medium. Cells were collected from 5 mL of the culture by centrifugation (3380×*g*, 2 min, RT). The cell pellet was washed twice with 1 mL of PBS (137 mM NaCl, 8.10 mM Na_2_HPO_4_, 2.68 mM KCl, and 1.47 mM KH_2_PO_4_), dissolved in 200 µL of PBS containing 50 µM FM4-64 (Thermo Fisher Scientific) and left at RT for 1 h. The washed cell pellet was cultured in 1 mL of YPD at 30 °C with agitation at 800 rpm for 1.7 h. The recovered cells were collected by centrifugation (3380×*g*, 2 min, RT), resuspended in 200 µL of PBS and observed. Fluorescence confocal images were obtained using a laser scanning microscope (Zeiss LSM800, Carl Zeiss, Jena, Germany) equipped with the 488-nm line or the 544-nm line of a diode laser and a 63 × 1.2 numerical aperture water immersion objective (C-Apochromat, 441777-9970-000, Carl Zeiss). The isolated vacuoles stored at − 80 °C were recovered at 4 °C and dissolved in 1 mL of PS solution [10 mM PIPES-KOH (pH 6.4), and 1.0 M d-sorbitol] and collected by centrifugation (16,000 × *g*, 0.5 min, 4 °C). The recovered vacuoles were suspended in 100 µL of SS solution [10 mM NaCl, 10 mM MgSO_4_·7H_2_O, 0.1 mM CaCl_2_·2H_2_O, and 0.2 M d-sorbitol]. FM4-64 was added at a final concentration of 50 µM and the suspension was left for 1 h. After centrifugation (16,000×*g*, 0.5 min, 4 °C), the collected vacuoles were resuspended in 100 µL of SS solution and observed as described above.

### Other assays

The cell concentration was determined by measuring the OD_600_ value using a Gene Quant 1300 spectrometer (GE Healthcare Life Sciences, Buckinghamshire, UK). The ATP concentration was measured by a luciferin-luciferase assay, as described previously [2].

### Supplementary Information

**Additional file 1:  Fig. S1.** Effect of dR expression on cell growth and intracellular ATP content. *S. cerevisiae* harboring vacuoles with or without dR were cultured at pH 7 and 4 (adjusted by HCl) for 18 h under light (100 µmol photons/m^2^/s) conditions. a The cell density (OD_600_). b The relative intracellular ATP content. Means and standard deviations are shown (*n *= 3).

## Data Availability

All dataset(s) supporting the conclusions of this article are included within the article.
