# Supplementary material for: Engineering yeast with a light-driven proton pump system in the vacuolar membrane
Source: Microb Cell Fact. 2024 Jan 3;23:4. doi: 10.1186/s12934-023-02273-1 (PMC10763269; doi:10.1186/s12934-023-02273-1)
Supplement: Supplementary file 1 — Additional file 1: Fig. S1. Effect of dR expression on cell growth and intracellular ATP content. S. cerevisiae harboring vacuoles with or without dR were cultured at pH 7 and 4 (adjusted by HCl) for 18 h under light (100 µmol photons/m2/s) conditions. a The cell density (OD600). b The relative intracellular ATP content. Means and standard deviations are shown (n = 3). [file 12934_2023_2273_MOESM1_ESM.doc]

**Supplemental Data**


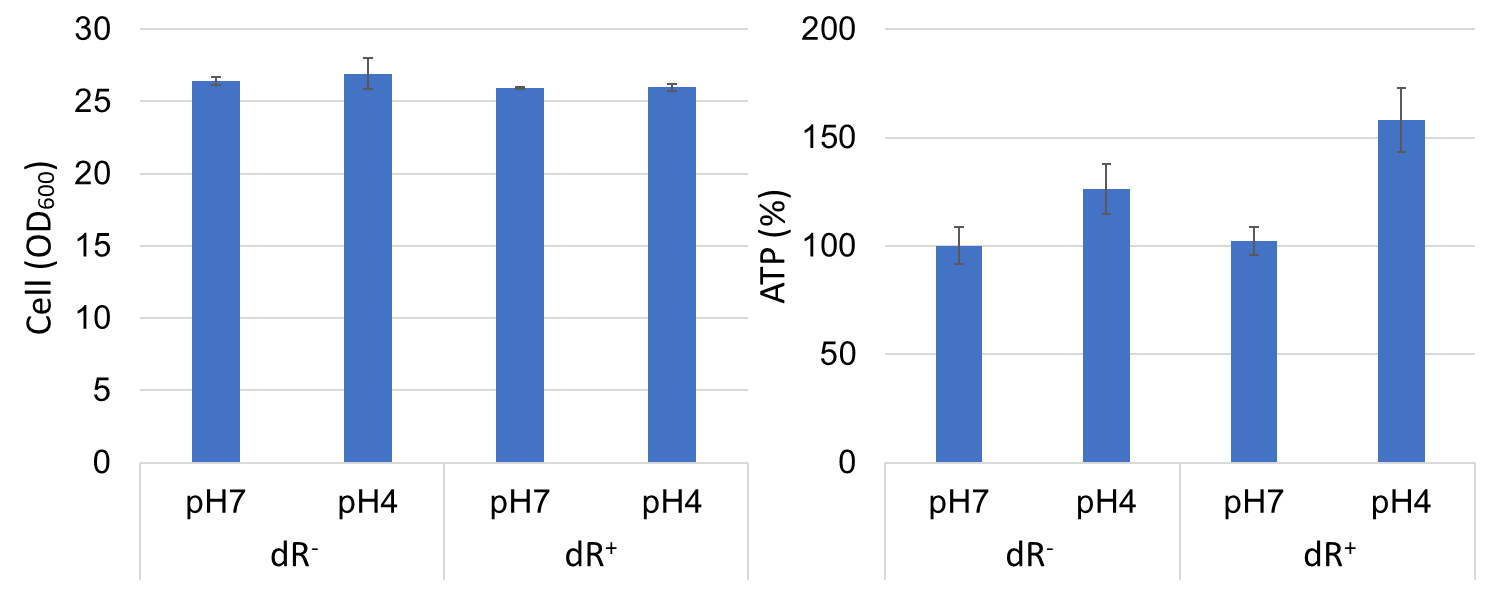


**Fig. S1** Effect of dR expression on cell growth and intracellular ATP content. *S. cerevisiae* harboring vacuoles with or without dR were cultured at pH 7 and 4 (adjusted by HCl) for 18 h under light (100 µmol photons/m^2^/s) conditions. **a** The cell density (OD_600_). **b** The relative intracellular ATP content. Means and standard deviations are shown (*n = 3*).
